# Electrocortical correlates of attention differentiate individual capacity in associative learning

**DOI:** 10.1038/s41539-024-00236-8

**Published:** 2024-03-18

**Authors:** Elsa Raynal, Kate Schipper, Catherine Brandner, Paolo Ruggeri, Jérôme Barral

**Affiliations:** 1 Brain Electrophysiology Attention Movement Laboratory, Institute of Psychology, University of Lausanne, Lausanne, Switzerland; 2 Institute of Sport Sciences, University of Lausanne, Lausanne, Switzerland

**Keywords:** Learning and memory, Human behaviour, Attention

## Abstract

Associative learning abilities vary considerably among individuals, with attentional processes suggested to play a role in these variations. However, the relationship between attentional processes and individual differences in associative learning remains unclear, and whether these variations reflect in event-related potentials (ERPs) is unknown. This study aimed to investigate the relationship between attentional processes and associative learning by recording electrocortical activity of 38 young adults (18–32 years) during an associative learning task. Learning performance was assessed using the signal detection index *d’*. EEG topographic analyses and source localizations were applied to examine the neural correlates of attention and associative learning. Results revealed that better learning scores are associated with (1) topographic differences during early (126–148 ms) processing of the stimulus, coinciding with a P1 ERP component, which corresponded to a participation of the precuneus (BA 7), (2) topographic differences at 573–638 ms, overlapping with an increase of global field power at 530–600 ms, coinciding with a P3b ERP component and localized within the superior frontal gyrus (BA11) and (3) an increase of global field power at 322–507 ms, underlay by a stronger participation of the middle occipital gyrus (BA 19). These insights into the neural mechanisms underlying individual differences in associative learning suggest that better learners engage attentional processes more efficiently than weaker learners, making more resources available and displaying increased functional activity in areas involved in early attentional processes (BA7) and decision-making processes (BA11) during an associative learning task. This highlights the crucial role of attentional mechanisms in individual learning variability.

## Introduction

Human beings exhibit variation across multiple domains, including physical characteristics, personality traits or cognitive abilities. Studying individual differences in cognitive abilities is of particular interest as it can shed light on the neural underpinnings of cognitive functions, and improve our understanding of cognitive disorders and their related possible interventions^1^. Among these abilities, associative learning is notable for exhibiting consistent and striking individual differences in performance^2–5^. The fundamental process underlying associative learning involves the creation of connections between two events, such as environmental stimuli and behavioral responses^6^. The formation of these connections is shaped by the presence or absence of reinforcement, and entails processing sensory information, attention, memory, and decision-making^7^. For instance, in a simple reinforcement task where participants receive a reward for pressing the correct button in response to a visual stimulus, participants first process the visual signal, mobilize their attention to identify the stimuli, store this information in memory, and decide to respond or withhold their response depending on the stimulus. The creation of these connections during the learning process has been shown to be greatly modulated by selective attention^8–12^ (for a review, see ref. ^9^).

Selective attention filters out irrelevant information and enhances sensory and perceptual processing of relevant features for the task at hand^13,14^. Behavioral studies have demonstrated that highly salient stimuli, thought to attract more attention because of their distinctiveness^15–17^ are learned more rapidly than less salient ones^18,19^. Additionally, instructing participants to direct their attention toward a selected set of stimuli (i.e., pairs of words) increased learning rates and the number of words recalled from this set^20,21^. Conversely, studies requiring participants to learn pairs of words under divided attention conditions showed impaired performance in memory compared to undivided attention conditions^8,22^. Studies using fMRI, even if not specific to associative learning, also confirmed a strong link between attention and learning. The investigations underscore the crucial role of attention in shaping the modulation of neural correlates throughout both memory encoding and retrieval processes^23–25^. Notably, studies indicate that attentional states significantly impact the activation patterns within key memory brain structures, such as the hippocampus, demonstrating an interdependence between attention and learning and memory^23,25,26^. While these findings provide compelling evidence for the importance of selective attention in associative learning, they primarily rely on group-level comparisons. Future research investigating how individual differences in selective attentional capacity influence learning rates within associative tasks can offer valuable insights and further solidify this hypothesis. However, few studies have been conducted in this regard, specifically examining the impact of selective attentional processes to individual differences. In recent studies^27–29^, eye-tracking methods—with pupil dilation and fixations as indicators of the intensity and duration of attention, respectively—were used during verbal paired-associates tasks. The authors were interested in examining whether individual differences in attention during the encoding phase were associated with differences in performance in the recall phase. Results showed that individual capacity to focus and maintain attention on stimuli was related to recall performance, even after controlling for working and long-term memory capacities^28,29^. In other words, participants who allocated more attentional effort when encountering the stimuli retained them better. These studies confirm a link between interindividual modulations of attention and learning performance at the behavioral level. To reach a more comprehensive understanding of this association, further investigations using cerebral correlates involved in attentional processing are warranted. Yet, a small number of studies have relied on non-invasive measures of brain activity, such as electroencephalography (EEG) or functional magnetic resonance imaging (fMRI) to investigate neural underpinnings of attentional processes and their relationship with individual differences in learning. Therefore, in the present research, we sought to strengthen this hypothesis using EEG.

EEG is particularly advantageous given its high temporal resolution, allowing capturing phenomena that occur in the range of dozens of milliseconds. This is especially valuable when event-related potential (ERP) components have been shown to be modulated by attention as early as 80 ms after the presentation of stimulus^30^. The P1 component, peaking between 80 and 130 ms after stimulus presentation and marked by a positive potential distribution over medial occipital scalp regions^13,31^, and the N1, a negative component peaking around 100 to 200 ms at parietal, central, and frontal scalp sites^32^ are the most commonly reported in relation to attention. Higher amplitudes of the P1/N1 complex were obtained for attended stimuli locations compared to unattended ones^33–36^. Similarly, higher amplitudes were recorded for correctly cued as compared to uncued stimuli^37–39^. Both the P1 and N1 components amplification are thought to reflect the perceptual enhancement of relevant stimuli and spatial locations with attention^13,30,31^. Additionally, the later P3 component, a parieto-central positivity wave occurring between 300 and 900 ms post-stimulus depending on the task^40–42^ has also been linked to attentional processes. This component is more specifically elicited in tasks requiring the discrimination and identification of targets^43^ and is thought to reflect attentional control and engagement, memory updating and evaluation and discrimination of stimuli^42–45^. Relative to associative learning, ERP studies often focused on differences before and after learning. For instance, post-training learned associations of names and human-like shapes were characterized by higher amplitudes of all components—including P1/N1 and P3—compared to pre-training unlearned associations^46^. When participants gradually learned the relationship between a cue and a target, larger amplitudes after learning were described in the N1 and P3 components at the cue presentation compared to before learning^47^. Wills et al.^48^ compared pairs of stimuli associated with less/more errors in the prediction of their outcomes and found that associations prone to prediction errors were learned faster and evoked a higher N1 amplitude. They interpreted this effect as an augmented visual discrimination and features enhancement of these stimuli. Eye-tracking in the same study showed that the stimuli associated with surprising outcomes had more viewing time (used as an index of the amount of attention directed to the stimuli) than less surprising ones, further suggesting that early attentional processes and associative learning are linked. No difference in P3 was found in this study, probably because ERP were analyzed only until 500 ms post-stimulus, while the P3 can be elicited until 900 ms post-stimulus^41^. To summarize, the P1/N1 components, and to a lesser extent P3, have been related to attentional processes and are modulated during learning.

The ERP analysis can help to elucidate neural mechanisms underlying learning and provide insight into how individual differences in specific abilities relate to learning success. However, few studies used these components as markers of attentional capacity while focusing on individual differences in learning. One exception is Abla et al.^49^, who approached this question by linking statistical learning capacity and ERP features. Statistical learning represents the capacity to extract regularities from the distribution of stimuli to distinguish patterns in these sequences. In this study, participants were exposed to continuous tone sequences, played in a random order, with no silent blanks between them. Subsequently, they were categorized into three (high-, middle-, and low-) learner groups, based on their recognition performance. Compared to middle and low learners, high learners exhibited larger N1 amplitudes, suggesting potential differences in attention allocation. The authors propose that high learners might allocate more attention than low learners toward both the underlying patterns within the stimuli and the hypothesized boundaries of the tone sequences, with particular emphasis on the onset. This attentional allocation would be the factor that differentiates learner groups. Although this study did not specifically investigate associative learning, it provides evidence that individual differences in electrocortical activity can provide insight into the neural mechanisms underlying learning.

The present study aimed to investigate whether attention-related EEG components are associated with individual differences during associative learning. To achieve this goal, based on previous studies on associative learning^50,51^, we created an associative learning task consisting of twelve abstract shapes that had to be associated with one of four colors (blue, green, orange, red) through trial-and-error. The difficulty of the task was calibrated to our sample population of young, healthy university students on account of the results of our pilot studies. The merit of this task is that it avoids ceiling effects observed in other psychometrically validated tasks, such as the Wechsler Memory Scale^52^ or California Verbal Learning Test (CVLT-II^53^) when used in non-clinical populations^54,55^. Another advantage of this task is that it allows for the computation of Signal Detection Theory indices. Despite its potential advantages over classical behavioral measures, the Signal Detection Theory (SDT) framework is not commonly used in learning studies. SDT allows for the estimation of the capacity of discrimination of relevant stimuli (*d’*) and response bias, providing a more comprehensive measure of learning performance. Using *d’*, a measure of perceptual sensitivity that quantifies the ability to distinguish signal from noise^56^, allows us to assess the impact of attentional processes on perceptual sensitivity. Specifically, as attentional processes are thought to improve the perceptual sensitivity of relevant features, *d’* allows us to assess their impact while minimizing the influence of response bias on task performance. Therefore, in our study, we chose *d’* as an indicator of performance in the associative learning task. At the brain level, we employed two complementary methods: Global Field Power (GFP) analysis and Topographical Analysis of Covariance (TANCOVA). GFP quantifies brain generator strength, providing a quantitative perspective on neural activity. However, it doesn’t reveal changes in the configuration of neural sources. In contrast, TANCOVA explores variations in scalp voltage topographies linked to experimental variables^57^. To ensure our findings were based on different cerebral activity sources rather than variations in source strength, we conducted TANCOVA on amplitude-normalized maps with GFP set to 1^58^. To pinpoint the sources contributing to these differences, we used source estimations. This approach allowed us to gain a comprehensive understanding of both the spatial distribution (with TANCOVA) and the magnitude of neural activity (with GFP), combining topographical and quantitative insights^59,60^. Based on the presented literature, we hypothesize that individual performance in the associative task, measured by the index *d’*, correlates with topographical maps within the time period of the P1/N1 complex, reflecting the modulation of attentional mechanisms. For the source generators, attentional processes that enhance the detection, discrimination and processing of stimuli linked to the P1/N1 complex are thought to involve a frontoparietal network^61,62^.

Accordingly, source generators linked to individual differences in learning are hypothesized to be part of this network. As the task requires discrimination and evaluation from the participant to distinguish if the stimulus is a target or a lure, a later modulation of the P3 component could be expected. As P3 generators are mostly linked to the frontoparietal and cingulate cortex^41^, individual differences related to this component are expected to be underlined by differences in activity within these regions.

## Results

### Behavioral data

The task was efficient at revealing individual differences given the large variability across individuals in correct responses (min = 48%, max = 88%, mean ± std = 72% ± 8%) and the large range in number of trials needed for the participants to reach the learning criterion imposed (min = 196, max = 501, mean ± std = 434 ± 89). It is important to note that 19 participants did not reach the criterion after the maximum number of trials allowed (i.e., 504). The *d’* index ranged from −0.09 to 2.32 (mean ± std = 1.19 ± 0.51) across participants. The average response time (mean ± std = 1235 ms ± 45 ms) exceeded the 1000 ms time window utilized for the ERP analyses making it improbable that motor-related potentials significantly influenced our results.

### Topographic consistency test

The TCT was applied to the pre-processed ERPs computed from 100 ms before to 1000 ms after the onset of the stimulus and showed significant and consistent topographies across the subjects at all time frames. The TANCOVA and GFP analyses were thus performed over the entire period.

### Topographic analyses of covariance and source estimations

**Fig. 1:**
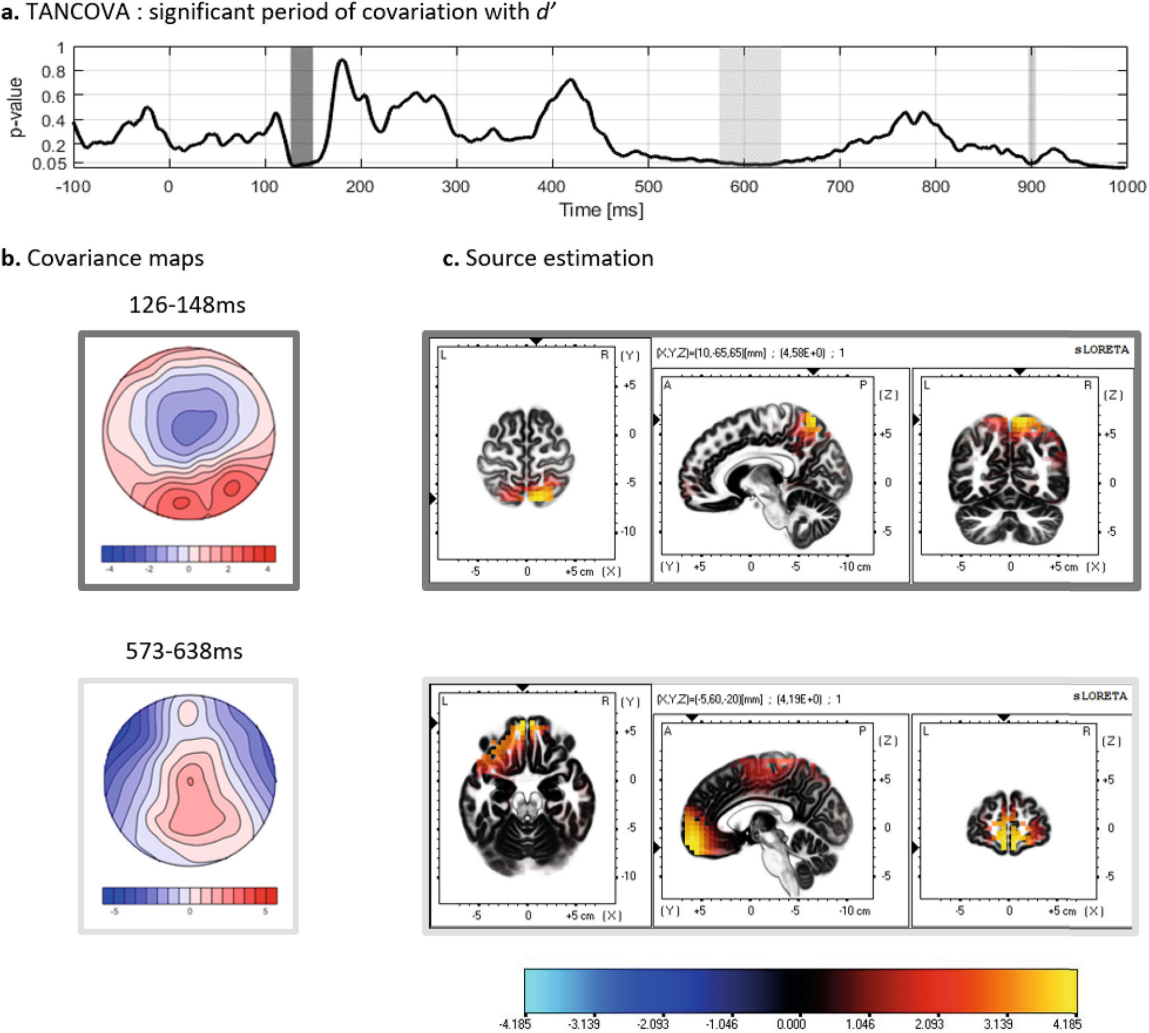
Results of the TANCOVA and sLORETA analyses computed using the ERPs during the associative task. **a** The *p* value of the TANCOVA is plotted as a function of time. The gray shaded areas highlight the time periods of significant covariation of ERP topographies and *d’*, between 126 and 148 ms (dark gray) and between 573 and 638 ms (light gray). The short significant period around 900 ms was not considered in the analyses due to its short duration (<20 ms). **b** The covariance maps observed from both significant periods 126–148 ms (upper panel) and 573–638 ms (lower panel). These covariance maps represent the topography associated with an increase of *d’*, while the topography associated with a decrease of *d’* would be the inverse topography. **c** sLORETA visualization of the underlying sources of the covariance maps showing activation in the precuneus (BA 7) during the first period (upper panel) and activation of the superior frontal gyrus (BA 11) during the second period (lower panel). The color scale depicts sLORETA value.

The TANCOVA revealed two significant time periods (Fig. 1a), the first at 126–148 ms and the second at 573–638 ms post-stimulus onset. A shorter period at 900 ms post-stimulus did not reach the 20 ms time criteria and was therefore not further analyzed. The covariance maps corresponding to these significant time periods are shown in Fig. 1b. The first significant period covariance map displays a topography marked by a negative potential at central sites, a positive potential at occipital sites, and a more diffused positive potential at fronto-temporo-parietal sites for increased *d’* (Fig. 1b, upper panel) (maximum value = 2.76 at electrode Oz; minimum value = −1.96 at electrode Cz). To estimate the brain sources of the significant covariations of ERP topographies with *d’*, source analyses were performed with sLORETA on the covariance map. Underlying sources for the first covariance map (Fig. 1c, upper panel) revealed an implication of the precuneus (BA 7), with the highest activation value at MNI coordinates X = 10, Y = −65, Z = 65. The second significant period shows a covariance map displaying a topography marked by a positive scalp potential distribution over central regions for increased *d’* (Fig. 1b, lower panel, with maximum value = 2.2 at electrode Cz; minimum value = −3.6 at electrode F7). The sources associated with this period computed with sLORETA are shown in Fig. 1c (lower panel) and include the superior frontal gyrus (BA 11), with the highest activation at MNI coordinates X = −5, Y = 60, Z = −20, and to a lesser extent, the medial frontal gyrus (BA 6).

### GFP analyses and source estimations

**Fig. 2:**
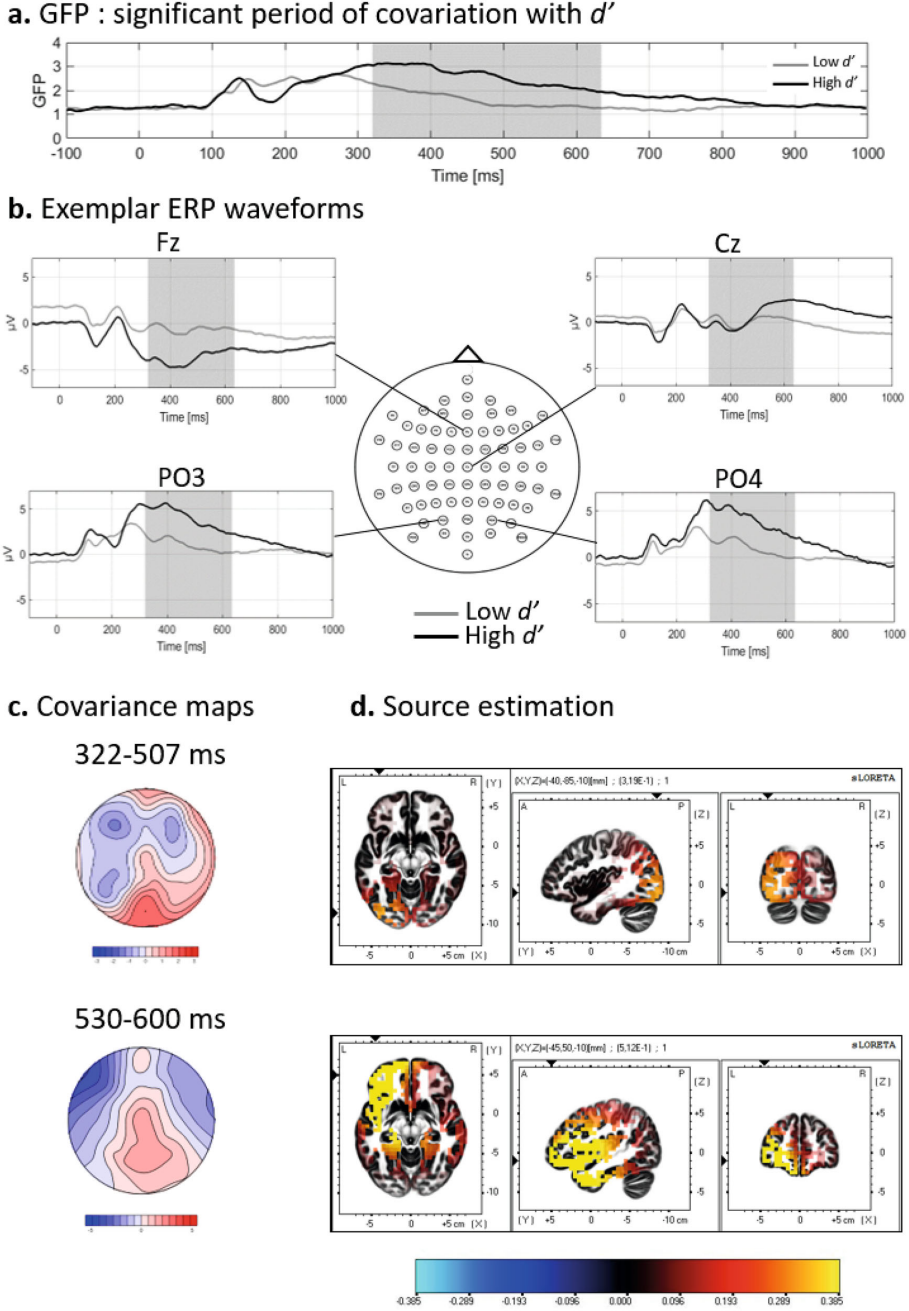
Results of the GFP and sLORETA analyses computed using the ERPs during the associative task. **a** The mean GFP value for 10 high *d’* participants (black line) and 10 low *d’* participants (gray line) are plotted as a function of time. The gray shaded area highlights the time period (322–634 ms) of significant covariation of GFP and *d’*. **b** Exemplar ERP waveforms (Fz, Cz, PO3 and PO4 electrodes) from the average of the 10 highest (black line) and 10 lowest *d’* (gray line) participants. The gray shaded area highlights the time period (322–634 ms) of significant covariation of GFP and *d’*. **c** The large GFP significant time period was divided in two periods revealing different effects. The covariance map for the first period at 322–507 ms is illustrated in the upper panel, while the lower panel displays the covariance map for the second period at 530–600 ms. These covariance maps represent the topography associated with an increase of *d’*, while the topography associated with a decrease of *d’* would be the inverse topography. **d** sLORETA visualization of the underlying sources of the covariance maps showing activation in the middle occipital gyrus (BA 19) for the first period 322–507 ms (upper panel) and in the middle and inferior frontal gyri (BA 11 and 47) for the 530–600 ms period (lower panel). The color scale depicts sLORETA value.

The GFP analysis revealed a significant positive association of the GFP with *d’* at 322–634 ms (Fig. 2a), indicating that stronger activation of active neural sources was associated with higher *d’*. Electrodes Fz, Cz, PO3, and PO4 are shown in Fig. 2b as exemplar waveforms from the mean of the 10 highest and 10 lowest *d’* participants. In order to further analyze this large time period, we had to make sure that it did not include different effects following one another in time. To control for this possibility, we divided the interval 322–634 ms into ~70 ms windows around time points where the explained variation was the highest, resulting in three periods, at 322–392 ms, 437–507 ms and 530–600 ms post-stimulus. The covariance maps for these three periods were submitted to spatial correlations between them to assess their similarity. The results indicated that the two first periods were very similar and could be merged into one (*r* = 0.92, *p* < 0.001), while the third period was not highly correlated with the other two (*r* = 0.59, *p* < 0.001 with the first one and *r* = 0.69, *p* < 0.001 with the second one) and thus reflected a distinct topography. The first resulting covariance map (between 322–507 ms) is shown in Fig. 2c (upper panel) and displays a topography marked by a positive scalp potential distribution over occipital sites, and a more diffuse negative potential over frontal regions for increased *d’* (maximum value = 2.04 at electrode Oz; minimum value = −1.39 at electrode F5). The underlying sources for this period (Fig. 2d) included the middle occipital gyrus (BA 19), with a maximum value at MNI coordinates X = −40, Y = −85, Z = −10. The second covariance map for the period between 530–600 ms displayed a topography marked by a posterior positivity (Fig. 2c, lower panel) (maximum value = 1.97 at electrode POz; minimum value = −3.4 at electrode F7). The underlying sources suggested for this period (Fig. 2c, lower panel) implicated the middle frontal gyrus (BA 11), with maximum MNI coordinates X = −38, Y = −37, Z = −8, and the inferior frontal gyrus (BA 47).

## Discussion

The objective of this study was to investigate the role attentional processes may play in individual differences in associative learning. We hypothesized that performance in an associative learning task would be related to topographical maps within the time period of the P1/N1 complex, reflecting the involvement of early attentional processes in associative learning abilities. We expected associated modulations in subregions of the frontoparietal network. As the task requires the participant to distinguish whether the stimulus is a target or a lure, a modulation of the P3 component with performance was also postulated, with generators in the frontoparietal and cingulate cortex.

ERP and source analyses revealed three main results. First, performance in the task was associated with a scalp topographic difference at 126–148 ms and related to the participation of the parietal cortex in participants with a better performance (increased *d’*). Second, performance in the task was linked to a topographic difference at 573–638 ms, overlapping with a difference of GFP at 530–600 ms, with associated source generators located within the frontal cortex for higher learners. Third, performance in the task was associated with a difference of GFP at 322–507 ms, attributed to a stronger participation of the occipital cortex for higher learners.

Using a data driven method, without a priori timing or location of effects, our approach yielded insights into individual differences in associative learning. The main result of this study confirmed our hypothesis that performance in the task and ERP scalp topographies significantly covaried during a time period that is typical of early attentional-related components. The resulting map illustrates the regions on the scalp where the amplitude of the ERP is proportional to the variations in *d’*. The significant covariance map between 126 and 148 ms, characterized by a positive occipital potential distribution for an increase in performance of learners (increased *d’*), is in line with the characteristics of the ERP P1 component^13,31^. Previous studies have shown a higher amplitude of P1 in cued or attention-orienting conditions^13,20,33,35^ or post-learning^46^, but our results reveal that the topography related to this component also varies across individuals who have different learning capacity. A higher P1 amplitude has previously been associated with enhanced perception of relevant stimuli and their location^30,38^. Therefore, it can be suggested that greater attentional involvement, along with an associated enhancement in the perception of relevant features of the task—for example, here, shape and color—may be linked to improved learning outcomes. This exciting result suggests that the temporal dynamics of early attentional-related components are closely associated with differences in associative learning abilities across individuals, thus underscoring the significance of visual attention processes in this context. Moreover, as the TANCOVA analysis had been normalized by the GFP, the significant covariation is likely due to the spatial distribution of the underlying active brain sources^57^. These sources, after localization of the topography’s generators, indicated an additional participation of the precuneus for participants exhibiting a higher *d’*, and more specifically in the right precuneus. This lateralized activation is congruent with previous findings suggesting that the right precuneus may be more specialized for attention, particularly in tasks involving target detection^63^. This lateralization is further supported by studies indicating greater right hemisphere activity for attention dominance^64^. This localization of sources in the precuneus is also consistent with previous fMRI studies that have shown a role for the precuneus in attention, and especially when directing attention in space^38,65,66^. More specifically, precuneus activity has been demonstrated to relate to several cognitive functions, including attention, working memory, episodic memory retrieval, and visual awareness^67^. Notably, the precuneus exhibits sensitivity to visuospatial attention, and its activity has been shown to predict subsequent memory for the location of stimuli, further highlighting its integral role in orchestrating cognitive processes associated with attention and spatial memory^23,62^. Specifically to associative learning, a MEG study^68^ required participants to observe pictures of natural scenes, and then associate them with colors by trial and error. The results reported that fast learners engaged a parieto-posterior network comprising the precuneus early after the presentation of the scene (0–200 ms), while slow learners engaged this network much later in the trial. The authors interpreted this activity as reflecting visuo-spatial processing, allowing faster learners to better represent the natural scene and its spatial elements. However, in this study, rather than differences of underlying processes, it was the timing of recruitment of the network that differed between participants. Related to differences in temporality, in a study exploring mental rotation^69^, the authors found that individual performance to the task corresponded to a P1 topography more present in better learners, but this difference was due to the P1 component seemingly lasting longer. This possibility cannot be excluded in our study, and future research should explore these components with complementary methods to investigate their temporal dynamics and distinguish between differences in the timing of the (same) processes involved or differences in the processes themselves.

Despite our hypothesis expecting a difference between individuals in the P1/N1 complex, our results indicate that topographic differences between individuals are only related to P1, as we did not find a frontocentral negativity topography similar to the N1 component. This finding opposes a previous study by Abla et al.^49^ that showed that processes reflected in the N1 component could underlie individual differences in learning, and with studies showing that N1 is modulated with associative learning^47,48^. It must be noted however that factors including experimental design, stimuli modality or intensity are known to influence ERP components^70–73^. As the study by Abla et al.^49^ used an auditory modality and a statistical task, results might not be generalizable to our study.

In addition to early attentional processes, this study explored later components, until 1000 ms after stimulus onset, as we also expected modulations in the P3 component. This was confirmed by a significant covariance between performance in the task, scalp topography and GFP around 600 ms, with covariance maps for an increase in performance in the learning task (*d’*) characterized by a central positivity with a posterior distribution, a topography classically associated with the P3. More specifically, this topography and its relatively late latency resembles the P3b, a subcomponent of the P3, with a more centroparietal topography than the P3 or P3a^40,43,45^. The P3b component is thought to reflect the effortful processing of task-relevant events, indicating the match between incoming stimuli and the voluntarily maintained attentional trace of the task-relevant stimulus^42,43,45^. It is often observed during tasks involving the evaluation and comparison of sensory stimuli^43,45^. It becomes prominent when a change in stimulus attributes is detected, leading to the engagement of attentional mechanisms for updating the neural representation of the stimulus context^43,74^. This process is thought to index memory storage operations, with P3b amplitudes related to the memory for previous stimulus presentations. In this context, the observation of a P3b topography associated with an increase in performance in a learning task is congruent, given that our task contains target stimuli, which moreover demand evaluation^43,75^. The difficulty of the task, coupled with the need to maintain stimuli in memory until feedback is provided after the response, aligns with the established role of P3b in reflecting effortful processing of task-relevant events and memory engagement. The increased P3b topography linked with an increase in performance in the learning task might suggest that better learners engage more effective attentional allocation and memory processes than lower learners.

However, given the controversy over the direct link between P3b and memory processes^75–77^, caution is warranted in interpreting the observed relationship with learning task performance. The debate over whether P3b reflects memory engagement or serves as a correlate of decision-making complicates the interpretation of the P3b topography in our study. Therefore, while the observed increase in P3b topography with performance may indicate improved attentional allocation, its interpretation should be nuanced in light of the ongoing discourse on the specific role of P3b in memory operations. This nuanced approach is further warranted by our source estimations results. Notably, our source estimations did not reveal significant activity in regions commonly linked to memory, such as hippocampal regions, and sometimes linked to the P3b component generation^78,79^. Indeed, the GFP covariance maps are estimated to originate from the left middle and inferior frontal gyrus (BA 11 and 6) and the topographic covariance map estimation indicated an origin in the middle and superior frontal gyri (BA 11 and 47), bilaterally. These regions are involved in multiple networks, but given the associated P3b topography, the timing and the specificity of our task, we assume that this activity reflects the involvement of the frontoparietal network^80^. This network is associated with cognitive effort in general, as a resource for general and diverse cognitive demands^81,82^. As our task is cognitively demanding because of its difficulty (19 participants failed to reach the criterion on correct response), the participation of these regions in better learners could suggest that the task requires the recruitment of resources that are less or not recruited by less good learners.

A complementary explanation is that activity in this network has been linked to the regulation of attention. In particular, the frontoparietal network is assumed to play a role in the top-down regulation of attention, possibly by guiding attention to remain focused on perceptual features relevant to the task in progress^83^. This is congruent with the P3b component proposed function in our study, where the regulation of attention to specific targets among stimuli could help better learners discriminate targets and lure in a complex task.

As stated previously, the results revealed an overlap between two distinct findings: first, a difference in GFP reflecting increased recruitment of certain regions; and second, a difference in topography indicating variation in the contribution of these underlying sources. These results seem to indicate that better learners exhibit stronger activation of these sources, with a similar initial process as lower learners, followed by a transition to another process resulting in differing underlying sources. As noted above, we were not able in this study to disentangle if these changes are due to a difference in the temporality of process, or a difference in the nature of the processes engaged. This question remains open and could be further explored in future studies.

An unexpected result in this study was the finding that performance in learning covaried with GFP, at the period of 322–507 ms post-stimulus. The associated topography was characterized by a positive distribution over occipital sites and a diffuse negative distribution over frontal areas. The source localization estimated this activity to originate from the left middle occipital gyrus (BA 19), meaning that a better performance was associated with increased activation of this region. As part of the visual associative cortex, this region has been linked to the visual capacity to discriminate visual stimuli, and especially shapes and colors^84,85^. In this process, attention could play a role of modulation, by enhancing the visual capacity of relevant features. The fact that the middle occipital gyrus is specialized in shapes and colors fits with the demanded associations of features in our task. The difference in GFP, but not in topography, suggests that all participants induce this process of visual discrimination, but it seems that better learners engage more resources^57^.

The source estimation from both our significantly different GFP periods indicated lateralization to the left. While functional differences between hemispheres are recognized, attentional processes are typically associated with increased activity in the right hemisphere^64^, contrasting with our findings. However, factors such as changes in experimental design, stimulus material, or memory processes are known to influence activity lateralization^86–90^. Alternatively, our task may exhibit an (involuntary) directional bias. Introducing a counterbalanced design, where half of the participants have the “yes” response button on the right and the other half on the left, or incorporating a task with mirrored stimuli, could potentially mitigate these effects. This consideration should be addressed in future research employing the same task.

Finally, the decision to separate the GFP analysis into two time windows is acknowledged as a chosen approach, representing the initial interpretation of the data with possible limitations. This decision was based on the extensive period of significant differences in GFP, with the goal to capture and describe the dynamics of electrical brain activity related to the task, while estimating that further subdivisions would have led to redundancy in our results. However, the large period or significant difference might indicate a lack of sensitivity in our analysis, and future studies may explore alternative approaches to address these limitations.

While acknowledging limitations in our lateralization findings related to potential design and task-specific factors, the core findings of our study provides important insights into the link between attentional mechanisms and individual differences in associative learning. Attention has long been known to play a crucial role in learning, and our study sheds light on the specific neural mechanisms that may underlie these differences. By using early ERP components, we present evidence that differences in early visual attentional processing could underlie individual differences in learning ability. Better learners present more of P1 topography, indicating an enhancement of visual processing modulated by attention. We also showed that a later component, with a P3b topography, differed between individuals, and suggest that better learners rely more on a frontal attentional network. These results have significant implications for our understanding of how attentional processes relate to individual differences in learning. Future research could investigate the generalizability of the present findings to other populations, such as older adults, or individuals with specific learning disorders. This will aid in determining whether the relationship between attentional processes and individual differences in learning is consistent across different populations. Additionally, to further understand the specificity of the present findings, future studies should also investigate the relationship between attentional processes and individual differences using different types of learning tasks. They could provide insight into whether the relationship between attentional processes and individual differences in learning is specific to visual associative learning or generalizable to other types of learning or presentation modalities. Moreover, future research could also investigate feedback loops in the learning process. Our results potentially indicate a sequence, beginning with early attentional processing in the precuneus, then progressing to object recognition and evaluations in the middle occipital gyrus, and ultimately involving frontal lobe attentional control and decision-making processes. This unfolding of cerebral events could illuminate the dynamic interplay during learning. However, the overlap between GFP and TANCOVA results in our study makes it challenging to pinpoint the exact sequence of events, emphasizing the need for dedicated research to untangle the complexities of these processes.

Overall, our study provides important insights into the neural mechanisms that underlie attentional processes and individual differences in associative learning, and highlights the importance of considering attentional abilities when investigating learning skills heterogeneity.

## Methods

### Participants

Forty right-handed healthy participants provided written, informed consent, and participated in exchange for course credits. They reported no history of neurological or psychiatric disorders or medication use and had normal or corrected-to-normal vision. Two participants were excluded due to the unavailability of more than 50% of their trials for data analysis. The final sample was composed of 38 participants (seven men; aged 22.3 years ± 4 years (mean ± SD), range 18–32 years old). The study was approved by the Ethics Committee of the Canton of Vaud (Switzerland; protocol No. 2019-02352) and was conducted according to the Declaration of Helsinki.

### Procedure and task

**Fig. 3:**
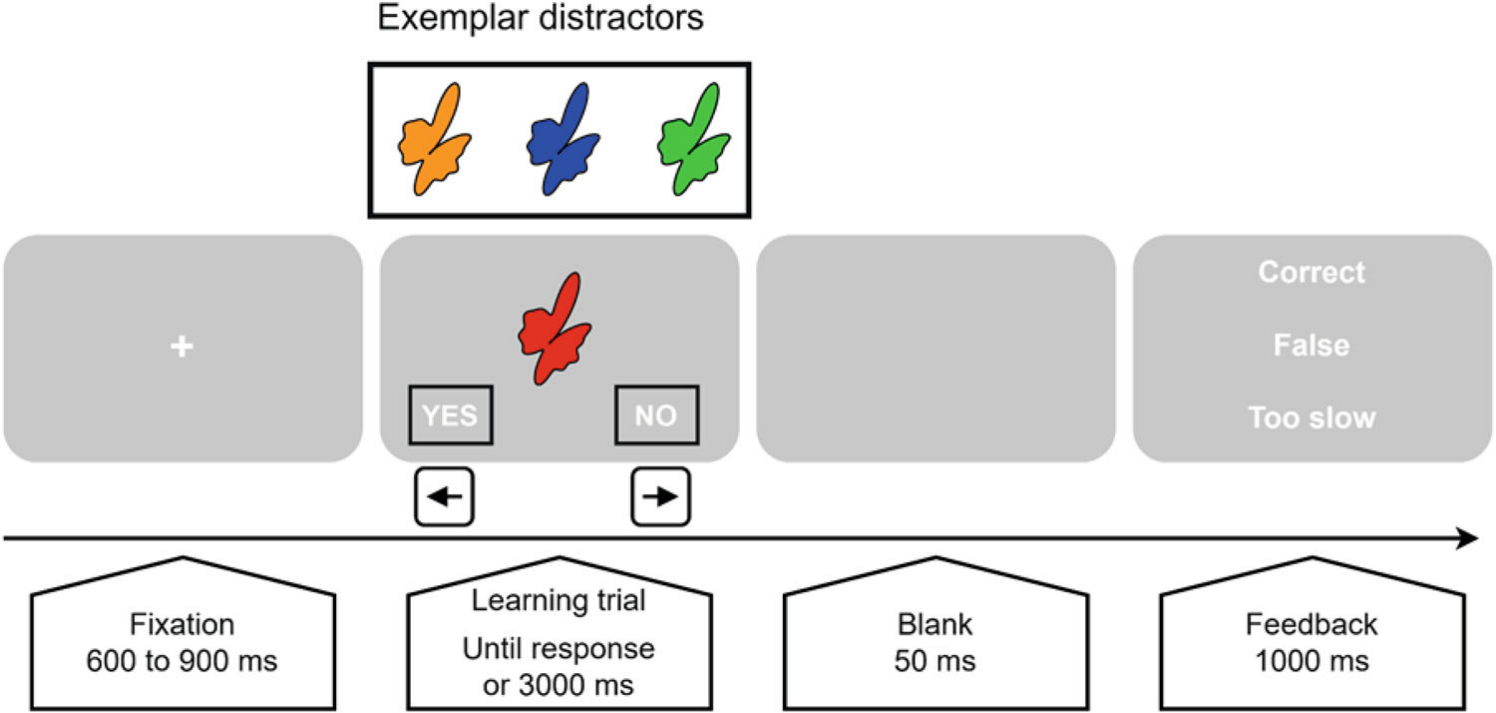
Experimental task design. Illustration of the sequence of the associative learning task with their respective presentation duration. The figure displays one exemplar stimulus (the “red” target stimulus) and its corresponding distractors (in this example, the same stimulus in “orange”, “blue”, or “green”) and the three different possible feedbacks (“Correct” was displayed after a correct response, “False” was displayed after an incorrect response, “Too slow” feedback was displayed after a response > than 3000 ms).

Stimuli consisted of 12 abstract, irregular and asymmetrical two-dimensional shapes created with MATLAB (Version 9.3.0 R2017b). To design targets, each stimulus was randomly assigned to one of four colors (blue, red, orange, or green), while the stimuli in the remaining colors were used as distractors. Stimuli were presented using PsychoPy v3.1.2.^91^ in a pseudo-random order, ensuring that each stimulus was seen 2 times in each block of 24 trials, once in the congruent color, once in a lure color. Lure colors were equally disseminated across the task. Stimuli were displayed in the center of 23-inch monitors at the size of 11° of visual angle vertically and between 8 and 11° horizontally, in combination with the yes/no response choice (Fig. 3).

Participants were comfortably seated in a sound-attenuating booth, in front of a monitor positioned at eye-level placed at a distance of 60 cm from their eyes. The task instructions were to gradually learn through feedback to associate each shape with its corresponding color and respond accordingly. In a familiarization session, 21 test trials with stimuli not included in the task were performed by the participant. Each trial began with a fixation cross for 600 to 900 ms, followed by a stimulus and response options (“YES” or “NO”) for 3000 ms or until a response was given. Participants endorsed or rejected the presented color-shape association by pressing the left (“YES”) or right (“NO”) arrow on the keyboard. Following a blank screen of 50 ms duration, a 1000 ms visual feedback informed the participant whether their response was correct, incorrect, or too slow if not made within the 3000 ms. Participants had to perform a minimum of 360 trials. Associations were estimated as being learned once responses within the last 48 trials reached the criterion of more than 95% correct. After the participant had reached the criterion, the task continued for 48 trials. As our task was quite demanding for the participant, we fixed the maximum number of trials to 504, after which the task stopped, even if the criterion was not reached. The task had a mean duration of 23 min depending on the number of trials participants underwent (ranging from 18 to 30 min at the maximum).

### Behavioral data

Behavioral statistical analyses were performed using the Statistical Package for the Social Sciences 27 (SPSS Inc., Chicago, Illinois, USA). Responses to the task were coded according to the Signal Detection Theory (SDT) contingency table as Hit (correct response to target), Miss (“no” response to target), Correct Rejection (CR, correct “no” response to non-target stimuli), or False Alarm (FA, “yes” response to non-target stimuli). The confusion matrix was constructed for each participant. The *d’* index was computed through a probit transformation (inverse function of the cumulative standard normal distribution, Φ-1) using the formula^56,92–94^:

1 $${d\text{'}}={\varPhi }^{-1}\left(\frac{{{\rm{Hit}}}}{\left({{\rm{Hit}}}+{{\rm{Miss}}}\right)}\right){-\varPhi }^{-1}\left(\frac{{{\rm{FA}}}}{\left({{\rm{FA}}}+{{\rm{CR}}}\right)}\right).$$

### EEG recording and ERP analysis

#### EEG acquisition and pre-processing

The electroencephalogram (EEG) was continuously recorded duringthe task at 1024 Hz using a 64-channel Biosemi ActiveTwo system with electrodes placed according to the international 10–20 system location. An active Common Mode Sense (CMS) electrode and a passive Driven Right Leg (DRL) electrode were employed as reference and ground, respectively, forming a feedback loop for the amplifier reference, and the electrode offset was kept within ±25 mV. EEG data pre-processing was performed using the Brain Vision Analyzer software (version 2.2.0.7383; Brain Products GmbH) using standard settings: data were down-sampled at 512 Hz, band-pass filtered (0.1–30 Hz), and vertical and horizontal eye movement artifacts were corrected with an independent component analysis (ICA,^95^). The event-related potentials (ERPs) were calculated by averaging the epochs from 100 ms before to 1000 ms after the onset of the stimulus for each participant. Trials with ±80 μV artifacts or with a difference between the maximum and the minimum exceeding 200 μV within 200 ms were automatically excluded. The remaining epochs were manually inspected to identify and eliminate those containing residual eye blinks and motor artifacts not removed by the ICA decomposition. Channels showing recurrent artifacts for prolonged periods were interpolated from neighboring electrodes, using linear splines interpolation of adjacent electrodes^96^. The average number (mean ± SD) of interpolated electrodes for participants was 1.68 ± 1.3 electrodes (2.63% of the 64 electrodes). EEG accepted epochs were then averaged by participants and recalculated against the average reference. As a conservative approach, no baseline correction was applied^60^. The average number (mean ± SD) of accepted epochs for all participants was 375.49 ± 69.62 epochs, corresponding to ~88% of the total number of epochs. Within the ~12% rejected, 40.5% were manually rejected, and 59.5% were automatically rejected.

#### Topographic consistency between subjects

All ERPs analyses were performed with the RAGU software (RAndomization Graphical User interface^57,97^; based on MATLAB (The MathWorks, Inc., Natick, MA, USA), and computed with 5000 randomization runs and a *p* threshold of 0.05. To identify time periods of consistent patterns of active sources (i.e., stable topographies) across participants, a topographic consistency test (TCT) was applied to the ERPs. The TCT is utilized to assess the consistency with which an event activates brain electrical sources across repeated measurements of event-related scalp field data—single-subject ERPs in our study. This analysis is typically conducted at the start of ERP analysis to identify time periods with a consistent association between the event and brain sources, and avoids drawing false conclusions if the period of interest is incorrectly selected^69,98^. Topographic and Global Field Power (GFP) analyses were performed on time periods showing consistent patterns of neuronal activation.

#### Topographic analyses of covariance (TANCOVA)

A TANCOVA was computed to identify periods of significant covariations between scalp topographies and the behavioral *d’* index^59,60^. With respect to classical ERP analyses^99^, topographical analysis has the advantage of being independent of the chosen reference and of not being influenced by expectations about timing and locations of specific components. This approach capitalizes on the concept that the presence of a source, active in proportion to an external variable (in our study, *d’*), contributes to a single topography that is integrated with the ERP in alignment with the external variable^100^. To extract the topography corresponding to the external variable at a specific moment, the covariance between the external variable and the potentials at each electrode is calculated. The resulting covariance map delineates the spatial distribution associated with generators that activate in proportion to the external variable at the specified moment in time. In other words, this analysis allows us to convey how the topography (in the form of the covariance map) is more or less present depending on the performance level in the task. Moreover, it allows to source the origin of neural changes by localizing the generators of the scalp topographies^60^. As a result, topographic changes are considered a direct interpretation of brain network activation because they follow changes in the configuration of intracranial generators^101^. To compute the TANCOVA, a covariance map (represented as a column vector, denoted as **V**) was computed for each time frame to investigate ERP topographies corresponding to the behavioral *d’* index. This covariance map was derived through a mathematical operation, specifically the matrix product **V** = **M**^T^*u, where **M**^**T**^ represents the transposition of the matrix of scalp potentials (**M**), and **u** is a column vector corresponding to the *d’* values of the individual subjects. This analytical process allowed the assessment of the covariation between ERP potential and *d’* at different time points (for more details, see ref. ^69^, section 2.5.1). The size of this covariation was quantified using the Global Field Power (GFP). The GFP corresponds to the standard deviation of the covariance map values and provides insights into the overall strength of the relationship between ERP potential and *d’* ^97^.

To assess the statistical significance of this covariation, non-parametric randomization tests were applied^100^. In each random iteration run, the presumed covariance between scalp potential and *d’* was eliminated by randomly permuting the elements of the *d’* vector. Then, the GFP of the newly computed covariance map was calculated and kept as the effect size. This procedure was repeated across 5000 randomization runs to create a distribution of effect sizes under the null hypothesis of no covariation between scalp potentials and *d’*. Finally, the real data was compared with the distribution of GFP values derived from the null hypothesis. The probability that the GFP in the observed covariance map was a random outcome was determined by calculating the proportion of instances where the observed GFP was either less than or equal to the GFP in the randomly generated covariance maps.

The TANCOVA was computed during the period validated by the TCT test on amplitude-normalized maps (GFP = 1) to reveal significant spatial differences independently of global field strength. To decrease the risk of false-positives, only significant differences at the threshold of *p* < 0.05 within a duration superior to ~20 ms were retained for further analyses^102^.

#### GFP analysis

An additional GFP analysis was performed to assess the relationship between the strength of all active neural sources and *d’*. GFP is calculated by taking the square root of the mean of the squared voltage values recorded across all electrodes at a particular time point^59^. In other words, it quantifies the average amplitude of EEG signals across all electrodes, effectively summarizing the overall magnitude of neural activity. The same criterion than for the TANCOVA—time interval validated by the TCT, duration superior to 20 ms and significance threshold (*p* < 0.05)—were applied.

#### Electrical source estimations

We estimated the origin of neural changes in the observed periods of significant covariation by submitting our data to a standardized low-resolution electromagnetic tomography method (sLORETA, version 20220427;^103^). This method uses a solution space composed of 6239 voxels, each with a volume of 5 mm × 5 mm × 5 mm, distributed within the gray matter of cortical and hippocampal regions of the Montreal Neurological Institute (MNI) template. Because the covariance map represents neural generator activity, it is possible to directly estimate the sources underlying the periods of significant covariation between ERP topographies and *d’*^100,104^.

### Reporting summary

Further information on experimental design is available in the Nature Research Reporting Summary linked to this article.

### Supplementary information

Reporting summary

## Data Availability

The datasets created and analyzed during the current study are available in the online repository: 10.5281/zenodo.10101332.
